# A Preliminary Validation of a New Surgical Procedure for the Treatment of Primary Bladder Neck Obstruction Using a Computational Modeling Approach

**DOI:** 10.3390/bioengineering8070087

**Published:** 2021-06-22

**Authors:** Michele Serpilli, Gianluca Zitti, Marco Dellabella, Daniele Castellani, Elvira Maranesi, Micaela Morettini, Stefano Lenci, Laura Burattini

**Affiliations:** 1 Department of Civil and Building Engineering, and Architecture, Università Politecnica delle Marche, Via Brecce Biance, 60131 Ancona, Italy; m.serpilli@univpm.it (M.S.); g.zitti@univpm.it (G.Z.); s.lenci@univpm.it (S.L.); 2 Department of Urology, IRCCS INRCA, 60124 Ancona, Italy; m.dellabella@inrca.it (M.D.); d.castellani@inrca.it (D.C.); e.maranesi@inrca.it (E.M.); 3 Department of Information Engineering, Università Politecnica delle Marche, 60131 Ancona, Italy; m.morettini@univpm.it

**Keywords:** urethral sphincter, tissue mechanics, computational biomechanics, bladder neck obstruction, ejaculation

## Abstract

A new surgical procedure for the treatment of primary bladder neck obstruction with maintenance of anterograde ejaculation is proposed. In place of monolateral or bilateral bladder neck incision, associated with a loss of ejaculation rate of up to 30%, the new surgical procedure consists of laser drilling the bladder neck with a number of holes and without muscle fiber disruption. The effect of this novel procedure has been studied numerically, with a simplified two-dimensional numerical model of the internal urethral sphincter, varying the position and the number of holes in the fibrotic region of the urethral tissue. Results show an improvement of the urethral sphincter opening by increasing the number of holes, ranging from about 6% to 16% of recovery. Moreover, a non-aligned position of holes positively influences the opening recovery. The concentrations of maximum principal strain and stress have been registered in the proximity of the interface between the physiologic and diseased sphincter, and in those regions where the radial thickness is significantly thinner. The effects on the first five patients have been included in the study, showing improvement in micturition, lower urinary tract symptoms, sustained ejaculatory function, and quality of life.

## 1. Introduction

Lower urinary tract symptoms (LUTS) in men are not solely correlated to benign prostatic hyperplasia. Men younger than 50 years often seek medical attention for their LUTS [1]. Primary bladder neck obstruction (PBNO) is the most frequent reason of voiding dysfunction in young men with an incidence of 50% [2]. First described by Marion in [3], this condition is characterized by inadequately opening the bladder neck and internal urethral sphincter during voiding, which leads to obstruction and voiding symptoms (hesitancy, strain to void, low and intermittent urinary flow). The actual pathogenesis of PBNO is still unknown, and the most accredited theories rely on structural change (hyperplasia and fibrosis) or hypertrophic muscle of the internal urethral sphincter, sympathetic nervous system dysfunction, and abnormal morphologic arrangement of the trigonal musculature [4]. Surgical treatment of PBNO is performed in case of urinary retention or LUTS not-responding to medical therapy. The traditional intervention is a monolateral or bilateral bladder neck incision at the 5 and 7 o’clock positions, 1 cm distal to ureteral orifices down to proximal to the verumontanum to completely disrupt the internal urethral sphincter [5]. Despite the excellent outcome in terms of obstruction relief and symptom improvement, bladder neck incision is associated with loss of ejaculation rate of up to 30%, which can be a problem in young men seeking fatherhood [6]. Furthermore, decreased ejaculatory volume or dry ejaculation might contribute to reduce orgasm and to increased levels of anxiety and depression [7]. The integrity of the bladder neck/internal urethral sphincter seems to be an essential part of anterograde ejaculation. Indeed, it has been demonstrated in vivo that the bladder neck in humans is closed during ejaculation with a contraction that lasts 30 s (see [8,9]). This contraction leads to an increase in bladder neck pressure up to 500 cm H2O that ends in anterograde ejaculation, [8].

The progress of novel surgical treatment procedures and the design of bioengineering medical devices are currently related to the investigation and knowledge of the macro-mechanical behavior of male urethral tissues and structure. Moreover, the development of computational models, corroborated by experimental characterization data, can improve the understanding of the biomechanical response of such tissues and be a support for surgical practices. Over the last few decades, the mechanical behavior of the majority of tissues and organs has been deeply studied (see, e.g., [10] for an extensive overview of the topic). Only recently, several works have been devoted to the biomechanical characterization of the lower urinary tract and urethral duct tissues from the experimental and computational points of view. The experimental investigations of the urethral biomechanics have been carried out through ex vivo tests on animal tissues. The choice of the animal urethra for ex vivo experiments strongly influences the mechanical response and, hence, a suitable animal model must be selected, with similar histomorphometric features to human urethra. In [11,12,13] sheep and porcine urethrae have been tested, proving that the swine specimens showed similarities to human ones in terms of functionality and mechanical behavior. In [14], Natali and co-authors provided relevant experimental results on horse urethral tissues, showing that stallions have comparable penis conformation to humans and represent a suitable model for ex vivo tests. Finally, in [15], membranous and spongy portions of urethrae of male cadavers have been sampled and tested on planar tension load/unload cycles in order to study their microstructure, anisotropy, and viscosity. Small animals such as rodents, rabbits, or dogs are not taken into account for their different penile size and anatomical structure.

The construction of a reliable computational finite element (FE) model allows us to understand the overall biomechanical behavior of the organic tissues and their possible interaction with biomedical stents and devices. An exhaustive research work has been carried out in [16,17,18,19,20,21], focusing on the overall mechanics of the urethral duct: the authors developed a numerical model, based on experimental data on a horse penile urethra and a theoretical constitutive modeling, to evaluate the biomechanical response of urethral tissues and structure. In [22], the proximal urethra deformation has been studied through the construction on a numerical model based on experimental results on rabbit urethral tissues. Finally, in [23], the authors investigated the mechanical, compositional and morphological characterization of the human urethra for the development on an engineered biomimetic tissue by means of an extensive experimental campaign. Hence, the development of a numerical model could help to evaluate the mechanical response of the sphincteric valve after the aforementioned surgical procedure.

The present work aimed at performing a preliminary validation through a computational modeling approach of a new surgical procedure for the treatment of primary bladder neck obstruction, with preservation of anterograde ejaculation. The computational model is developed in two stages: (i) the opening of the fibrotic internal urethral sphincter subjected to an imposed internal pressure is simulated; (ii) the opening of the fibrotic internal urethral sphincter, after a new surgical procedure (see below), is numerically evaluated. A comparison between the strain and stress fields before (urethra with PBNO) and after surgery is performed, highlighting the efficacy of such surgical procedure in terms of the sphincteric opening deformation recovery. Moreover, a parametric analysis is carried out by varying the position and the number of holes in the fibrotic region of the urethral tissue, produced by the surgical laser. The numerical model will allow us to assess the reliability of the new surgical procedure for the treatment of PBNO. It is important to remark that the computational model is based on the clinical evidence coming from the particular adopted surgical procedure developed by the urologists of our research group.

## 2. Materials and Methods

### 2.1. Surgery Description and Clinical Study Design

We developed a new surgical approach of PBNO with the idea of preserving the bladder neck/internal urethral sphincter anatomy and contraction, maintaining an anterograde ejaculation together with micturition improvement. We assumed that it might be enough relaxing the internal urethral sphincter and widely opening the bladder neck musculature through its full thickness drillings with a number of holes and without muscle fibers disruption. The latter can be achieved only using a small laser fiber. The suitable laser for this full thickness drilling was identified with the Thulium: yttrium-aluminum-garnet laser (TL). Indeed, TL operates at a 1940–2013 nm wavelength, in a continuous wave mode, which enables accurate cutting of the tissue, with no tissue disruption due to its optical penetration of only 0.2 mm (see [24]).

The surgical procedure was performed using 0.9% saline solution and a continuous flow 26 Ch endoscope (Karl Storz, Tuttlingen, Germany), mounted with a 12° optic and with a separate operative channel for the fiber. Laser energy was transmitted through an 800-micron, front-firing laser fiber. The laser setting was 30 W (RevoLix DUO 120W, LISA Laser products, Katlenburg, Germany). Surgery started with enucleation of the transitional zone 1 cm proximal the verumontanum to preserve both ejaculatory ducts and the musculus ejaculatorius [25]. Once the internal urethral sphincter fibers have been exposed, 6–7 holes were performed at the level of the bladder neck (see below Results and Supplementary Video S1). An 18 Ch urethral catheter was placed at the end and left inside for 24 h.

Inclusion criteria were LUTS lasting for at least one year and not responsive to medical therapy, International Prostate Symptom Score (IPSS) > 8, maximal urine flow rate (Qmax) less than 15 mL/s, and willing to maintaining anterograde ejaculation. Exclusion criteria were previous and concomitant surgery of the lower urinary tract. Age, prostate volume, IPSS, Male Sexual Health Questionnaire-Ejaculatory dysfunction (MSHQ-ED), Qmax, intraoperative, and postoperative data were gathered. Early complications were considered within 30 postoperative days.

Both IPSS and MSHQ-ED are two validated and international questionnaires that are widely used in clinical practice. IPSS is a self-administered questionnaire consisting of 8 items that assess LUTS (urgency, frequency, nocturia, incomplete emptying, intermittency, weak stream, and straining to void) and quality of life associated with LUTS [26]. The overall score ranges from 0 to 35, with a higher score displaying more severe LUTS, and from 0 to 5 for quality-of-life item. MSHQ-ED is also a self-administered questionnaire consisting of three ejaculatory function items (force, volume, and frequency,) and one bother/satisfaction item, [27]. The scores range from 0 to 5 for ejaculatory function items with a lower score meaning more severe ejaculatory dysfunction and from 0 (no problem) to 4 (extremely bother) for bother/satisfaction item. Postoperative follow-up visits were scheduled at 1, 6, and 12 months after surgery.

The first 5 men meeting inclusion criteria were enrolled in this pilot study. The study was approved by our local Ethical Board (DGEN 421/2017). Signed informed consent was obtained from all patients.

### 2.2. Numerical Model of the Fibrotic Internal Urethral Sphincter

**Figure 1:**
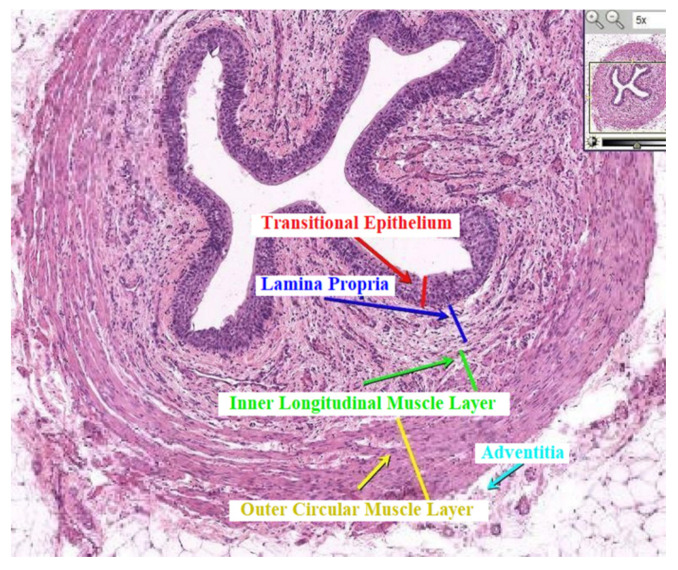
Histological section of human urethral sphincter.

The experimental evidence and the image analysis during surgery (see Supplementary Video S1) allowed to reconstruct the urethral sphincter section, suffering from PBNO, which is mainly constituted by two parts: an upper sane region, preserving its full mobility and deformability, and a lower fibrotic region, stiffer and less deformable than the top part. A specific histological section is selected for the development of the model, see Figure 1.

**Figure 2:**
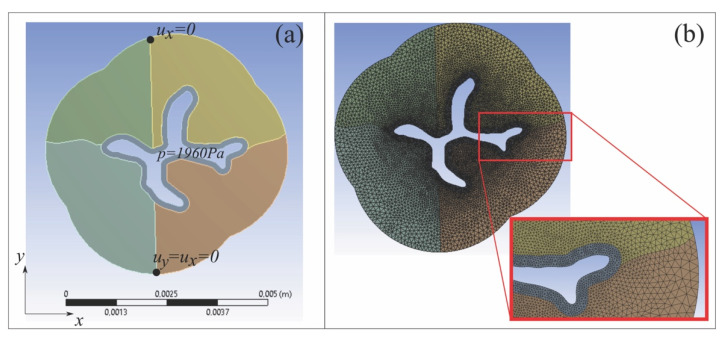
Simplified two-dimensional geometry, reproducing the internal urethral sphincter. (**a**) Sketch of the domain, with boundary conditions. (**b**) Numerical implementation of the geometry, with mesh.

The definition of a solid and numerical model is based on a simplification of the internal urethral sphincter geometrical features. The domain consisted of a two-dimensional geometry, reported in Figure 2a, that reproduced the anatomy of the section of the internal urethral sphincter (Figure 1). The model is composed by a layer of dense connective tissue, surrounding the urethral duct (transitional epithelium), and a spongy stratum (lamina propria) divided into four parts, connected along with two bounds 1 mm long each. The two bottom parts are the ones that can be fibrotic. The use of a simplified two-dimensional geometry was functional to the purposes of the present research and, in particular, to provide a mechanical justification to the recovery of opening deformation of the fibrotic sphincter, after the aforementioned surgery procedure.

The domain was constrained with the assignment of the displacements (u_x_, u_y_) at two points, as described in Figure 2a, which allowed the non-constrained opening of the sphincter due to the pressure *p* = 1960 Pa prescribed on the internal boundary (see Figure 2a). Perfect interface conditions among the different portions of the urethra section have been considered.

Model and mesh configurations were defined through the procedures of computational mechanics. The computational model was developed assuming incremental mesh refinement.

**Figure 3:**
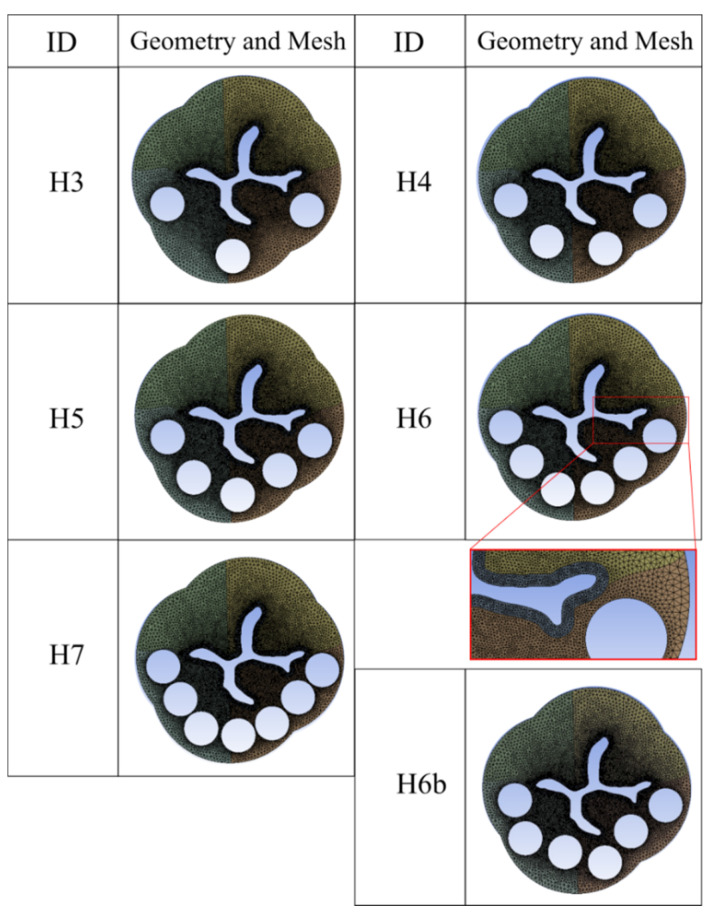
Meshed geometries of the domains for the numerical implementation, for all the studied perforated configurations.

The two-dimensional solid model of the urethral sphincter was discretized, and meshed with ANSYS PLANE183 elements, with a maximum edge size 10^−4^ m (see Figure 2b). The mesh size was refined in the thick layer of dense connective tissue and in the surrounding of such layer (see Figure 2b), with maximum edge size 5·10^−5^ m. The mesh elements were defined by three nodes, with two translations at each node. Large deflection was considered in the mesh mapping and plane stress element behavior was assumed. When holes were introduced in the geometry, the mesh was also refined in the surrounding of the holes (see Figure 3). The nonlinear adaptive mesh method was applied to the perforated region, which automatically re-meshed the model when excessive element distortion (mesh angle above 160°) occurred. To take into account the nonlinear behavior with large deformations of the urethral tissues, a 5-parameters Mooney–Rivlin material was employed, as suggested in [22]. The material coefficients of the epithelium dense connective tissue and spongy stratum have been found by fitting the exponential constitutive law (cf. [14], Equation (2)), using the set of parameters defined in [20], for horse penile urethra. To simulate the bladder neck fibrosis, in absence of experimental data, the initial stiffness of the diseased region was considered of the same order of magnitude of the epithelium internal tissue. The strain energy W for a 5-parameters hyperelastic Mooney–Rivlin model is defined by

(1) $$W=C_{10}\left(I_{1}-3\right)+C_{01}\left(I_{2}-3\right)+C_{20}{\left(I_{1}-3\right)}^{2}+C_{02}{\left(I_{2}-3\right)}^{2}+C_{11}\left(I_{1}-3\right)\left(I_{2}-3\right)+\frac{1}{d_{1}}{\left(J-1\right)}^{2},$$

where C_ij_ and d_1_ represent the material coefficients, I_1_, I_2_, and J denote the first and second Green deformation tensor invariants, and the volume ratio, respectively.

The Mooney–Rivlin coefficients for the epithelium, spongy and fibrotic parts are reported in Table 1.

The 5-parameters Mooney–Rivlin model also constituted a good fitting of experimental results presented in [23] for human male urethra. The hyperelastic formulation takes into account the typical features of soft biological tissues, namely, the quasi-incompressibility and nonlinearity; on the other hand, it neglects other complex behaviors, such as anisotropy and time-dependent phenomena. Nonetheless, this particular constitutive law is suitable for providing a preliminary assessment of the described surgical approach efficacy, through the evaluation of the sphincteric opening recovery (see, also, [22]).

### 2.3. Numerical Evaluation of the Effect of the Surgical Procedure

The opening of the fibrotic internal urethral sphincter, after the new surgical procedure, is numerically evaluated by considering configurations with a number of holes located in the fibrotic part ranging from 3 to 7. The holes had a radius of 0.5 mm and were uniformly distributed on a curve in the fibrotic part of the domain. The actual position of the holes during surgery is determined according to the patient anatomy and fibrosis extent. Each simulation was identified with the ID = Hn, with n being the number of holes. For the case H6, the position of the holes was varied with another different configuration, namely, a configuration with staggered holes, identified with ID = H6b.

All the different configurations are shown in Figure 3, where the meshed geometries of the domains for the numerical implementation are reported.

For comparison with the physiological condition, a domain without holes and characterized by sane material was considered. The corresponding simulation was identified by the ID = P0. Test P0, H0, and H6 has been run with different values of the pressure applied in the internal bound, ranged from *p* = 196 Pa up to *p* = 2584 Pa, with intervals of 196 Pa.

The cross-sectional area A has been used to evaluate the loss of opening capacity with respect to the no-holes fibrotic configuration:

(2) $$\delta_{l}=\frac{A\left(\mathrm{Hi}\right)-A\left(P0\right)}{A\left(H0\right)},$$

where A(P0) and A(Hi) represent the open cross-sectional area of the sane and fibrotic configurations, respectively. The opening deformation recovery has been evaluated as the difference of the open cross-sectional area with holes A(Hi) and with no holes A(H0), divided by A(H0):

(3) $$\delta_{r}=\frac{A\left(\mathrm{Hi}\right)-A\left(H0\right)}{A\left(H0\right)}$$

## 3. Results

The variation of the cross-sectional area of the urethral internal lumen is considered the result of the main interest for the aim of the present study, focused on the opening of the valve. The values of cross-sectional area A of the urethral duct for each configuration are reported in Table 2.

The ranges of maximum principal stress and maximum principal strain are reported in Table 3. For some representative tests, the mapping of these results has been considered in the following subsections.

### 3.1. Different Number and Position of the Holes

As reported in Table 2, the major loss occurs in the configurations without holes and corresponds to about 45%. The loss decreases with the increase of the numbers of aligned holes, up to the lowest loss, found in the configurations with six and seven holes, equal to 32.96% of loss. This corresponds to a deformation recovery of 11.87% with respect to the physiological condition. Results in Table 2 show also that the position of holes produces positive effects on the opening loss and recovery. Indeed, the non-aligned staggered configuration H6b presents an increase of opening and an improved deformation recovery.

**Figure 4:**
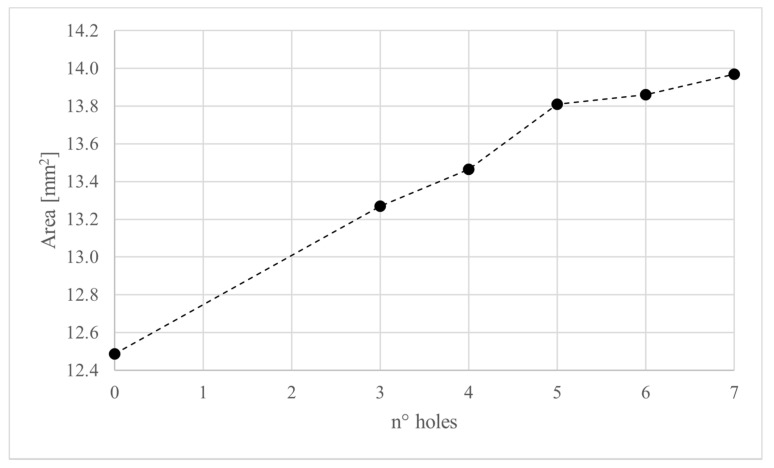
Variation of internal lumen area with respect to the number of holes, considering the cases H3, H4, H5, H6, and H7.

Figure 4 shows the variation of the internal lumen area with respect to the number of holes, considering the cases H3, H4, H5, H6, and H7.

**Figure 5:**
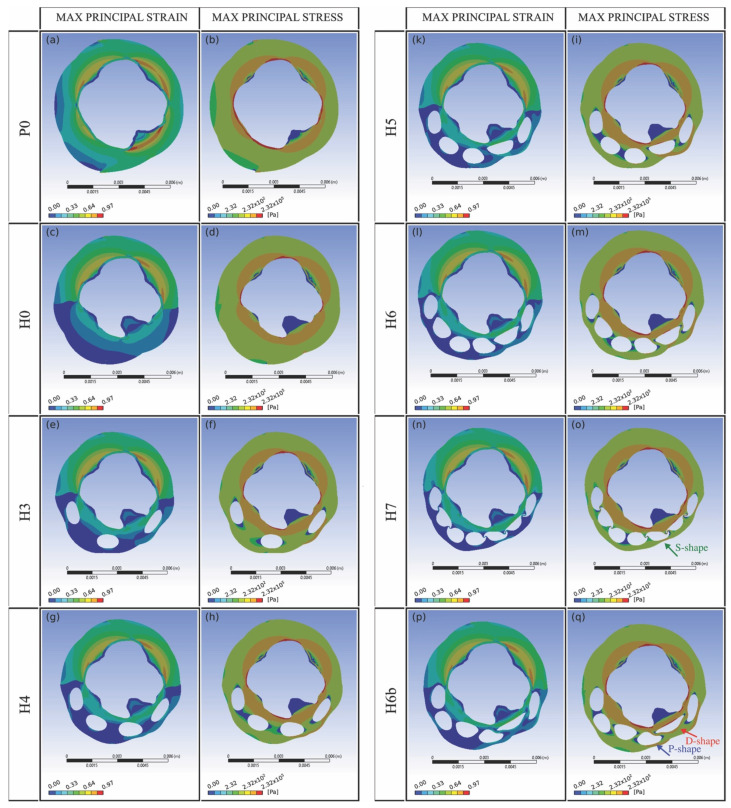
Deformed configurations: maximum principal strain in linear scale (**left panel**: subfigures (**a**,**c**,**e**,**h**,**k**,**l**,**n**,**p**)) and maximum principal stress in logarithmic scale (**right panel**: subfigures (**b**,**d**,**f**,**g**,**i**,**m**,**o**,**q**)).

Figure 5 reports the deformation of all the configurations, with maps of the maximum principal strain and maximum principal stress.

### 3.2. Effects of Intraluminal Pressure

**Figure 6:**
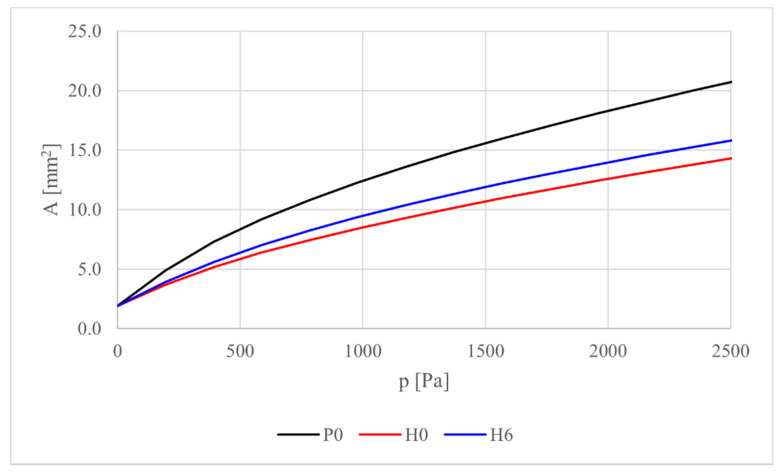
Variation of the lumen area for configurations P0, H0, and H6 with respect to the intraluminal pressures.

The variation of the lumen area for tests P0, H0, and H6 with different intraluminal pressures are reported in Figure 6.

### 3.3. Clinical Results

Five consecutive patients with a clinical diagnosis of PBNO who met inclusion criteria were included in the study. Table 4 shows patients’ characteristics. Mean age was 46 ± 12.7 years. Mean prostate volume was 25.6 ± 4.39 mL. Surgery was completed in all patients and mean surgical time was 22 ± 5.7 min. Mean delivered energy was 14.60 kJ. All patients were able to void in the second post-operative days and were discharged home. Two patients suffered from early complications: one urinary tract infection and one acute urinary retention due to blood clots. All patients maintained anterograde ejaculation 1-year after surgery. Follow-up visits showed improvement in micturition, LUTS and QoL, and sustained ejaculatory function (Table 5). One patient experienced acute urinary retention 8 weeks after surgery.

## 4. Discussion

The developed computational framework allows the investigation of the biomechanical opening behavior of the internal urethral sphincter structure, suffering from PBNO. The mechanical response is evaluated before and after the aforementioned surgical procedure, consisting of drilling a certain number of holes through the whole thickness of the membrane, without muscle fiber disruption. FE analyses were performed by assuming different geometrical configurations, varying the position and the number of holes in the fibrotic region of the urethral tissue, as shown in Figure 3.

The numerical results show an improvement of the urethral sphincter opening after perforation, which approaches the physiological condition P0 by increasing the number of holes within the fibrotic region. The loss of opening capacity *δ*_l_ shows a negative decreasing trend, passing from −44.83%, for H0-configuration, to −29.18%, for H7-configuration, while the opening deformation recovery *δ*_r_ presents an increasing trend, from 6.26% of the H3-configuration to 11.87% of the H7-configuration (Table 2). Thus, the removal of the stiffer fibrotic tissue, drilling an increasing number of holes, helps to make the material seemingly “looser”, allowing a partial improvement of the opening deformation. This speculation was confirmed by improvement in micturition in all patients who showed, during follow-up, meaningful higher Qmax and a drop in IPSS scores (Table 5). Moreover, Table 2 shows that a different position of holes positively influences the opening recovery, passing from 10.99% of the aligned holes configuration (H6) to 15.65% of the staggered holes case (H6b). The non-aligned configuration provided a loss of mechanical and geometrical symmetry, yielding to an enhancement in terms of opening recovery

The map of maximum principal strain and stress fields (see Figure 5) shows that stress and strain concentrations are mainly located within the inner annular region of the dense connective tissue. The pick values of principal strains and stresses are registered in correspondence of the contact region between the physiologic and diseased sphincter where the urethral tissue presents a significant reduction in radial thickness.

Figure 5 also shows a more marked formation of circular struts in configuration P0, corresponding to the maximum principal strain and stress. The insertion of holes modifies the urethra strain and stress states and, thus, the position of the ring-shaped struts. In particular, the drilling of a relevant number of holes (for instance, H5 and H6 cases) promotes the development of the annular strut within the fibrotic part, close to the internal lumen. While the maximum principal strain and stress tend to extend and distribute to a wider region for the H0 and H3 cases.

A higher number of holes (H5, H6, H7 cases) may enhance the structural compliance of the fibrotic region. This effect is confirmed by the significant distortion of the holes in the deformed configuration, assuming D-, P-, or S-shaped profiles, indicated in Figure 5o and Figure 5q with the red, blue, and green arrow, respectively.

**Figure 7:**
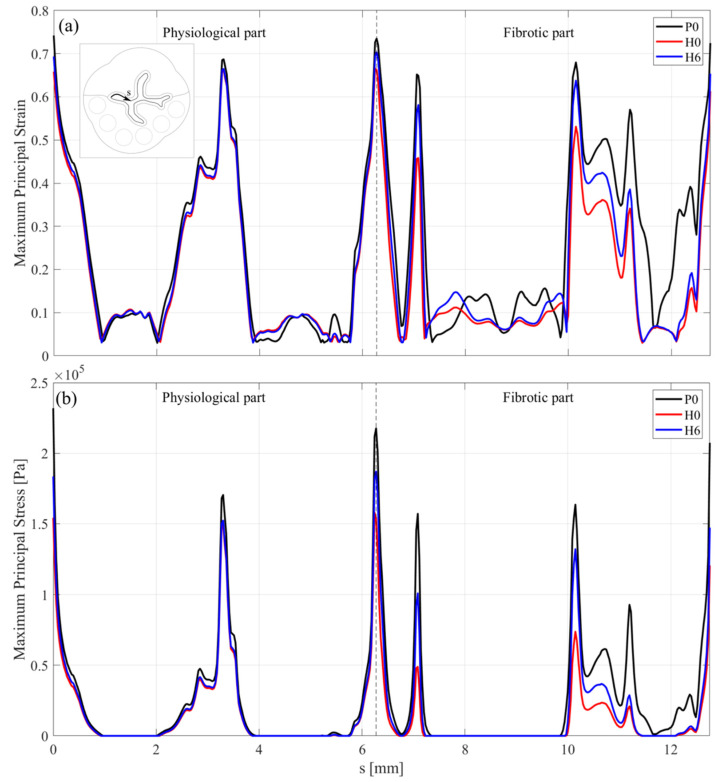
Maximum principal strain and stress along the intraluminal boundary for tests P0, H0, and H6: (**a**) physiological part; (**b**) fibrotic part.

As an example, the maximum principal strain and stress along the intraluminal edge for tests P0, H0, and H6 are reported in Figure 7, for the physiological and fibrotic parts. The curvilinear abscissa runs clockwise along the internal curve, outlining the epithelium inner boundary.

The above diagrams confirm that the peak values of the maximum principal strain and stress have been registered especially where the radial thickness is smaller. The highest principal stress can be found in proximity of the fibrotic interface. As expected, the case H6 is included between the completely sane urethra case (P0) and the diseased case (H0).

As concerns the sphincter response subjected to different intraluminal pressure, Figure 6 reports a nonlinear growth of the overall opening displacement as a function of the increasing inner pressure. These results are coherent with those obtained in [22] for rabbit urethra. As expected, the H6-case shows an intermediate behavior included between the physiological configuration P0 and the fibrotic configuration with no holes H0.

## 5. Conclusions

A new surgical procedure for the treatment of PBNO has been proposed, consisting of drilling the bladder neck with a number of holes and without muscle fiber disruption. A simplified numerical model of the internal urethral sphincter has been used for preliminary validation of the procedure, testing different configurations of the holes.

Numerical results showed that the drilling of holes improves the urethral sphincter opening, which increases with the number of holes. The use of a staggered configuration could positively help the opening recovery. The concentration of maximum principal strain and stress have been registered in the proximity of the interface between the physiologic and diseased sphincter and within the stiffer epithelium region, this suggests avoiding the drilling of holes in such regions in order not to compromise the valve integrity. Moreover, Figure 7 confirms that the holes must be placed where the urethral tissue is thicker, preventing eventual stress concentrations and, thus, local ruptures.

Finally, the insertion of a relevant number of holes (H6–H7) yields to an enhancement of the surgical procedure efficacy since the removal of much more fibrotic tissue promotes a more prominent deformation and distortion of the remaining material.

The feedback from the first five patients confirms the expected improvement in micturition, LUTS, sustained ejaculatory function and quality of life.

The accuracy of the present results can be considered satisfactory, in spite of the limitation that pertains to the two-dimensional numerical model configuration. This preliminary approach is also justified by the relevant computational effort determined by the high nonlinearity of the problem, related to material, geometry, and contact conditions.

Another limitation was the fact that this work is a pilot study, thus involving a small number of enrolled patients. However, the low rate of complications and long-lasting functional results were promising and allowed us to carry on a further clinical study.

## Figures and Tables

**Table 1:** Material constants for the 5-parameters Mooney–Rivlin material model.

Material Constants	C_10_ [Pa]	C_01_ [Pa]	C_11_ [Pa]	C_20_ [Pa]	C_02_ [Pa]	d_1_ [Pa^−1^]
Dense connective tissue	3030.83	2142.94	−1.431× 10^5^	59,059.00	82,911.00	8.00 × 10^−7^
Spongy tissue	89.12	82.60	−4737.35	2064.50	2953.61	8.00 × 10^−7^
Fibrotic tissue	891.18	825.97	−47,373.50	20,645.00	29,536.10	8.00 × 10^−7^

**Table 2:** Results of numerical simulations with different numbers and positions of holes in the fibrotic part.

ID	A [mm^2^]	Loss δ_l_ [%]	Recovery δ_r_ [%]
P0	18.09	-	-
H0	12.49	−44.83	-
H3	13.27	−38.57	6.26
H4	13.47	−37.00	7.83
H5	13.81	−34.25	10.58
H6	13.86	−33.84	10.99
H7	13.97	−32.96	11.87
H6b	14.44	−29.18	15.65

**Table 3:** Ranges of maximum principal stress and maximum principal strain.

ID	Maximum Principal Stress [Min-Max Pa]			Maximum Principal Strain [Min-Max]		
P0	0	-	213,950	0.01074	-	0.97004
H0	0	-	157,910	0.00250	-	0.87389
H3	0	-	178,790	0.00595	-	0.87539
H4	0	-	181,310	0.00237	-	0.87609
H5	0	-	185,710	0.00239	-	0.87787
H6	0	-	187,130	0.00058	-	0.87678
H7	0	-	203,870	0.00158	-	0.87641
H6b	0	-	196,710	0.00302	-	0.87976

**Table 4:** Pre- and post-operative patients’ characteristics.

Patient	Age, Years	IPSS ^1^	QoL ^1^	Q-Max ^1^, mL/s	PV ^1^, mL	Length of Surgery, Minutes	Number of Holes	Delivered Energy, kJ ^1^	30-Day Complication
#1	45	14	5	6.9	23	20	6	12	UTI ^1^
#2	58	20	4	9.3	30	30	6	10	
#3	58	21	5	11	30	20	7	19	
#4	27	21	6	9.7	20	15	6	10	Haematuria with catheterization
#5	46	10	5	7	25	25	7	22	

^1^ IPSS: International Prostate Symptom Score QoL: Quality of Life. Qmax: maximal urine flow rate. PV: prostate volume. kJ: Kilo Joule. UTI: urinary tract infection.

**Table 5:** Follow-up visits.

Patient	1-m ^1^ IPSS	1-m QoL	1-m Q-max, mL/s	1-m MSHQ-ED ^1^	6-m IPSS	6-m QoL	6-m Q-max, mL/s	6-m MSHQ-ED ^1^	12-m IPSS	12-m QoL	12-m Q-max, mL/s	12-m MSHQ-ED ^1^
#1	6	1	21	14	5	1	22.7	13	4	1	20	13
#2	10	2	16.6	13	4	0	15.3	12	3	0	16	12
#3	5	2	20	15	3	1	19.6	14	3	0	21.8	13
#4	2	1	16	12	2	1	15.4	11	3	1	18.1	12
#5	7	0	14	14	4	0	18.6	13	3	0	17.6	13

^1^ 1-m: 1 month; MSHQ-ED: Male Sexual Health Questionnaire-Ejaculatory dysfunction.

## Data Availability

The data presented in this study are available on request from the corresponding author.

## Supplementary Materials

The following are available online at https://www.mdpi.com/article/10.3390/bioengineering8070087/s1, Video S1: Surgery video.
